# Features of the metabolic syndrome and subclinical atherosclerosis in patients with cerebrotendinous xanthomatosis: An augmented risk for premature cardiovascular disease

**DOI:** 10.3389/fgene.2022.997069

**Published:** 2022-09-27

**Authors:** H. Cohen, S. Hassin-Baer, A. Shaish

**Affiliations:** ^1^ The Bert W. Strassburger Metabolic Center, Chaim Sheba Medical Center, Tel-Hashomer, Ramat Gan, Israel; ^2^ Sackler Faculty of Medicine, Tel-Aviv University, Tel Aviv-Yafo, Israel; ^3^ The Movement Disorders Institute and Department of Neurology, Chaim Sheba Medical Center, Tel-Hashomer, Ramat Gan, Israel; ^4^ Achva Academic College, Arugot, Israel

**Keywords:** cerebrotendinous xanthomatosis, low-density lipoprotein cholesterol, coronary artery disease, cholestanol, atherosclerosis, carotid intima-media thickness, metabolic syndrome

## Abstract

**Background:** Cerebrotendinous xanthomatosis (CTX) is a rare lipid storage disease, caused by deficiency of sterol-27-hydroxylase. Xanthomatous lesions in numerous tissues, and an elevation of cholestanol levels, characterize the disease. Its natural course is progressive neurologic deterioration, leading to premature death. Chronic treatment with oral chenodeoxycholic acid (CDCA) reduces cholestanol levels. Occurrence of premature atherosclerosis has been described in CTX in an unknown mechanism.

**Aim:** The aim of the current work was to evaluate the potential metabolic abnormalities and preclinical vascular changes in Israeli CTX patients.

**Methods:** Ten subjects with CTX were studied. Features of the metabolic syndrome were evaluated, and carotid intima media thickness (cIMT) was measured in the common carotid arteries.

**Results:** All patients were diagnosed with CTX, and all received treatment with CDCA, which resulted in normalization of their plasma cholestanol levels. At the conclusion of the follow up, risk factors for CVD and features of MS were present in all the patients and in three patients, cIMT was higher compared to control subjects.

**Conclusion:** Cardiovascular risk factors and premature vascular changes exist in young CTX patients and proper assessment should be implemented with preventive measures to reduce the risk of atherosclerotic cardiovascular disease in CTX patients.

## Introduction

Cerebrotendinous xanthomatosis (CTX) is a rare autosomal recessive lipid storage disease, caused by mutations of the CYP27A1 gene, resulting in deficiency of sterol-27-hydroxylase (CYP27). CYP27 is a mitochondrial cytochrome P450 enzyme, present in most cells in the body, with a key role in gap between hepatic bile acid synthesis (Nie et al., 2014), as it catalyzes steps in the oxidation of sterol intermediates that form bile acids. Normally, the ideal substrates for mitochondrial 27-hydroxylation are 7α-hydroxylated intermediates in bile acid synthesis (Björkhem 2013).

The biochemical hallmark of CTX is impaired synthesis of chenodeoxycholic acid (CDCA) from cholesterol, that results in elevated levels of plasma and bile cholestanol (Nie et al., 2014). Xanthomatous lesions in numerous tissues, including tendons, lens, brain, and additional tissues characterize the disease (Moghadasian et al., 2002). There is considerable variability in disease onset, developmental manifestations milestones, presence of intellectual deficiency or learning disabilities, later neurological deterioration, and systemic involvement, even among patients within the same family and genotype. The natural course of CTX is progressive neurologic deterioration from childhood through adulthood, leading to diffuse damage of the central, and peripheral nervous systems and eventually to premature death. Neurological deterioration due to cerebellar, pyramidal, and extrapyramidal system involvement, as well as additional cognitive decline, psychiatric symptoms, epileptic seizures, and peripheral neuropathy usually become evident in the second or third decades of life. Common non-neurologic manifestations of CTX include infantile-onset diarrhea, childhood-onset cataracts, osteoporosis, repeated bone fractures and respiratory insufficiency (Nie et al., 2014; Moghadasian et al., 2002; Moghadasian 2004; Kuriyama et al., 1991; Patni and Wilson, 2020). Chronic treatment with oral CDCA reduces cholestanol synthesis and lowers its plasma levels. Timely treatment, started early in life, may halt, or even prevent the neurological progression of CTX, and has the potential to reverse some neurological deficits (Nakamura et al., 1991; Verrips et al., 2020; Yahalom et al., 2013; Tao et al., 2019). Nevertheless, delayed diagnosis and therapy as well as decreased compliance and availability of the drug, remains a major problem in CTX (Yahalom et al., 2013; Yunisova et al., 2019). Primary neurological involvement is the principal concern in patients with CTX; nonetheless, substantial occurrence of premature vascular involvement has also been described, with different clinical manifestations of cardiovascular disease (CVD) in more than 10% of patients with CTX (Tada et al., 2018; Souto et al., 2020; Valdivielso et al., 2004).

In a publication from 1991, describing several patients with CTX, nearly half of them presented with atherosclerotic lesions on the coronary angiogram, suggesting a high prevalence of vascular changes in CTX (Kuriyama et al., 1991). The exact mechanism leading to early onset arteriosclerosis in this disease is unknown (Björkhem and Boberg 1998). Specific abnormalities in the lipoprotein profile were not identified in CTX, and cholesterol levels usually are within normal limits (Fujiyama et al., 1991). In a case report from 2004, a young patient was described with a myocardial infarction, lacking the classic risk factors for premature arteriosclerosis, except for mild mixed dyslipidemia and elevated apolipoprotein B levels (Valdivielso et al., 2004). Furthermore, the enzyme 27-hydroxylase, which is the malfunctioning enzyme in CTX, has additional roles in cells such as macrophages, and endothelial cells and is additionally involved in the process of the transport of cholesterol from the peripheral tissues to the liver (Babiker et al., 1997). Metabolic changes in high-density lipoprotein (HDL) contribute to the premature atherosclerosis as defects in HDL functionality and cholesterol efflux capacity are associated with of subclinical atherosclerosis in young and healthy subjects and in older and CVD patients (Hunjadi et al., 2020). Hence, decreased levels or dysfunction of the HDL particle in CTX may lead to alternation in the process of reverse cholesterol transport and is a potential mechanism for the accelerated atherosclerosis in CTX (Babiker et al., 1997; Von Bahr et al., 2002).

Carotid artery intima media thickness (cIMT) is non-invasive ultrasound guided technique of cardiovascular risk stratification and it is utilized for preclinical assessment of early atherosclerotic changes (Stein et al., 2008; Liu et al., 2020). Routine use of cIMT in CTX is not practiced, though in a publication describing a young CTX patient, carotid ultrasound facilitated the recognition of preclinical atherosclerosis (Burnett et al., 2001).

The metabolic syndrome (MS) is a cluster of metabolic manifestations and risk factors for diabetes and CVD (DeFronzo and Ferrannini, 1991) and the most used definition is the one of the National Cholesterol Education Program (NCEP) Adult Treatment Panel III (ATP III) (Alberti et al., 2009). Within the general Israeli population, in a recent large cohort of 230,639 participants, the prevalence of obesity, abnormal blood pressure, and Type 2 diabetes mellitus has increased dramatically throughout the years (Twig et al., 2019).

Additionally, studies have demonstrated a substantial relationship between fatty liver disease and CVD mortality (Ghouri et al., 2010). Elevated levels of liver function markers, gamma-glutamyltransferase (GGT), alanine aminotransferase (ALT), aspartate aminotransferase (AST) and alkaline phosphatase (ALP), have been associated with an augmented risk of CVD (Li et al., 2016; Pahwa et al., 2021; Rahmani et al., 2019).

There are two major clusters of CTX in Israel, one is in Jews of North African decent and the second is in the Druze, a small Middle Eastern religious sect from the north of Israel and each patient group has distinct mutations. In Jews from Moroccan origin, a deletion of thymidine in exon 4 and guanosine to adenosine substitution at the 3′ splice acceptor site of intron 4 of the gene have been found. There is an additional mutation in Jews of Algerian origin, which is a cytosine to thymidine transition at cDNA position 1037 leading to a threonine to methionine substitution at residue 306. In the Druze a CYP27 a deletion of cytosine in exon two results in a frameshift leading to a premature termination signal (Yahalom et al., 2013; Leitersdorf et al.,1993; Falik-Zaccai et al., 2008; Reshef et al., 1994).

As the assessment for features of the metabolic syndrome and cIMT are not used in the routine management of CTX patients, the aim of the current work was to evaluate the prevalence of MS and vascular changes in CTX in an attempt to elucidate the mechanism leading to the phenomena of premature CVD.

## Materials and methods

### Subjects

Ten CTX subjects were recruited at the outpatient clinic of the Movement Disorders Institute and at the Bert W. Strassburger Metabolic Center Outpatient Clinic at the Chaim Sheba Medical Center, Israel from January 2008 to January 2022. All patients had exhibited elevated plasma levels of cholestanol before starting therapy with CDCA, several years ago.

### Molecular genetics

All patients were of North African Jewish descent and were either homozygous or compound heterozygous for either one or two of the known mutations described in Jewish families of North African descent.

### Metabolic syndrome

Diagnosis of the MS in adults was done according to the National Cholesterol Education Program (NCEP), Adult Treatment Panel III (ATP III) (Alberti et al., 2009). Presence of any three out of the five criteria qualifies the definition of MS: abdominal obesity, waist circumference ≥102 cm in men and ≥88 cm in females, serum triglycerides (TG) ≥150 mg/dl or drug treatment for elevated TG, serum HDL cholesterol <40 mg/dl in males and <50 mg/dl in females or drug treatment for low HDL cholesterol. Blood pressure ≥130/85 mm Hg or drug treatment for elevated blood pressure or fasting plasma glucose (FPG) ≥100 mg/dl (or drug treatment for elevated blood glucose).

### Liver function markers

Following an overnight fast alanine aminotransferase (ALT), aspartate aminotransferase (AST) and alkaline phosphatase (ALP) were evaluated. Presence of hepatic steatosis was evaluated by liver ultrasound scan.

### Carotid artery intima-media thickness

Patients underwent a color-coded duplex examination of neck vessels using a 10 MHz linear array ultrasound (Hitachi medical corporation, Tokyo, Japan). IMT was evaluated on the common carotid arteries (CCAs) over approximately 1.5 cm proximal to the flow divider, according to standardized guidelines. IMT was measured at the thickest plaque-free point on the near and far walls with a specially designed computer program. Mean CIMT values from the far walls of the right and left common carotid arteries are reported (Liu et al., 2020). The control group for the IMT measurements, in patients less than 30 years old consisted of fifty-five (33 females) healthy normo-cholesterolemic patients aged 18–30 years, who underwent carotid IMT measurements at the Bert W. Strassburger Metabolic Center, between the years 2007–2014. Mean bilateral carotid IMT measurements in the healthy controls was 0.49 ± 0.059 mm (mean ± SD). The normal reference for patients aged 30–40 years was less than 0.65 mm and less than 0.75 mm for the age range of 40–60 years (Stein et al., 2008).

### Ethics committee

Ethical approval for the study was obtained from the ethics board of the Chaim Sheba Medical Center, Tel-Hashomer, Israel number 8210-21 SMC.

## Results

The baseline clinical, metabolic and biochemical characteristics of the ten patients included in the study are summarized in Table 1. None were smokers and none reported additional clinical ASCVD risk factors, symptoms, or imaging findings at the initial visit. Family history of MS or CVD was present in two of the patients at baseline.

**TABLE 1:** Baseline Biochemical and clinical characteristics of CTX patients (Features of the metabolic syndrome in bold).

No.	Gender	Follow up (years)	Age at DX^a^ (years)	Age at 1st visit	Blood pressure (mmHg)	BMI (kg/m_2_)	AST/ALT IU/L	Fasting glucose (mg/dl)	TC (mg/dl)	HDL (mg/dl)	TG (mg/dl)	Cal LDL cholesterol (mg/dl)	Non HDL-cholesterol (mg/dl)	^b^Number of metabolic aberrations
1	M	14	13	19	126/71	**29**	23/31	90	219	47	**168**	127	172	3
2	F	14	16	40	118/79	24.5	24/28	96	188	50	72	117	138	2
3	F	11	14	38	125/70	20	20/34	87	170	53	58	109	117	1
4	F	8	21	34	**135**/80	23	**46/60**	92	256	**43**	**171**	179	213	3
5	F	3	10	33	125/80	22	31/17	97	198	57	83	124	141	2
6	M	13	6	14	**132**/82	**29**	25/30	84	164	48	113	93	116	4
7	F	2	30	40	**140**//73	**28**	30/22	83	266	47	**234**	172	219	3
8	M	10	10	29	**143**/84	23	17/25	83	118	**37**	59	70	81	2
9	M	7	16	34	121/81	22.8	18/24	89	242	44	100	178	198	2
10	M	7	13	42	126/75	**25.5**	23/34	98	113	42	**169**	138	71	3
Mean ± SD		8.9 ± 4.3	14.9 ± 6.7	32.7 ± 6.7	S-128.1 ± 7.2	24.6 ± 3.1	25.7 ± 8.5	89.9 ± 5.7	193.4 ± 53.6	46.8 ± 5.7	122.7 ± 59.6	130.7 ± 36.7	146.6 ± 352.6	
					D-77.5 ± 4.9		29.4 ± 11.8							

a Age at diagnosis and start of CDCA, treatment.

b End of follow up, Normal range AST-0-37 IU/L, ALT 0-37 IU/L.

M, Male; F, Female; DX, diagnosis; m, meter; BMI, Body mass index; Kgs, Kilograms; ALT, Alanine aminotransferase; AST, aspartate aminotransferase; IU, international units; MS, metabolic syndrome; SD, standard deviation; S, systolic; D, diastolic; Cal, calculated; L, liter

All patients were diagnosed with CTX as children or adolescents, and one patient was diagnosed at the age of 21 years (14.9 ± 6.7 years, mean ± SD). All patients received treatment with CDCA (250 mg three times per day) that resulted in normalization of their plasma cholestanol levels (Data not shown). While neurological stabilization and steadiness was obtained in patients number 1–7, gradual deterioration was observed in patients 8–10, as described in our previous publication presenting the neurological consequences (Yahalom et al., 2013).

At the initial assessment, one patient presented with full criteria of the MS and six patients had features of the partial MS and obesity. During the mean follow up of 8.9 ± 4.3 years (mean ± SD) four additional patients fulfilled the required number of abnormalities for MS. Liver function markers of alanine aminotransferase (ALT), aspartate aminotransferase (AST) were above the normal range in one patient at presentation, and five patients developed altered liver function during follow up and ultrasonic features of fatty liver in the liver ultrasound scan. The mean time for the appearance of the metabolic abnormalities was 3.5 ± 2.2 (mean ± SD) years from diagnosis.

Hypercholesterolemia and elevated plasma LDL-cholesterol are not part of the five features of the metabolic syndrome, though they are as a well-known risk factor for CVD. Elevated levels of fasting plasma LDL-cholesterol >100 mg/dl were present in eight patients through the follow up period. At the conclusion of the follow-up, risk factors for CVD and features of MS were present in all the patients.

**FIGURE 1:**
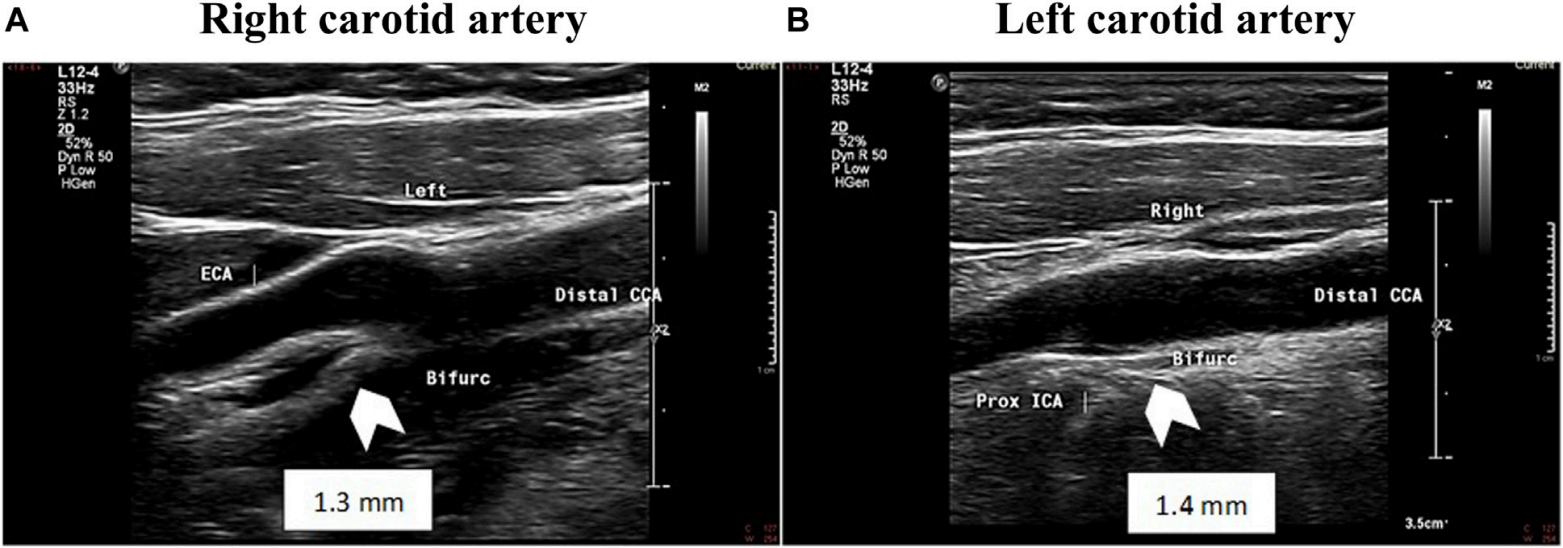
Carotid Intima media thickness. Ultrasound image showing measurement of near and far wall IMT in the distal 1 cm of the common carotid artery and increased carotid IMT of the common carotid artery (1.3 mm; 1.4 mm) of patient 2. White arrowhead -indicating increased carotid IMT. CCA, common carotid artery, ICA-internal carotid artery, Bifurc-bifurcation, mm-millimeter. **(A)** Right carotid artery, **(B)** B .Left carotid artery.

In two patients, the initial cIMT was higher in comparison to the normal range of their age group, with accelerated increment within 7 years in the patient number 2. In the third patient, though the initial IMT was within the normal range, atherosclerotic changes developed within 9 years (Table 2; Figure 1). To date there are no clinical events of atherosclerotic cardiovascular disease.

**TABLE 2:** Imaging characteristics of the patients- Carotid intima-media thickness measurements and carotid Doppler tests.

Patient	Age at cIMT (years)	Mean cIMT (mm)^a^
1	19	1.0 mm
2	45	1.1 mm
	53	1.4 mm
3	38	0.4 mm
	47	Carotid plaque LICA-25%

a Mean bilateral carotid IMT, measurements healthy controls −0.49 ± 0.059 mm.

Abbreviations: cIMT, Carotid intima-media thickness; LICA, left internal carotid

## Discussion

CTX is a rare inherited metabolic disorder; with a relatively high estimated prevalence in Israel with a carrier frequency of 1:80 in Jews of North African decent (Leitersdorf et al., 1993; Falik-Zaccai et al., 2008). We report here, a series of young patients with CTX, with features of the MS, hypercholesterolemia and pre-clinical atherosclerotic vascular changes. Remarkable disturbances of glucose metabolism were not demonstrated. Even though, the incidence of obesity and its metabolic consequences has increased in the Israeli population in the last two decades (Twig et al., 2019), the elevated proportion of CTX patients with dys-metabolism exceeds this tendency and appeared in all of the patients in the study.

Premature vascular involvement and clinical CVD manifestations have been described globally in CTX and may be attributed to the fact that CVD is the leading cause of death in most developed countries (Minelli et al., 2020). We propose that MS and its features developed as a related phenomenon to the CTX, resulting from lifestyle modifications of an early onset chronic illness, or due to the alternations in bile acid metabolites subsequent to the inborn error of metabolism (Duell et al., 2018; Sekijima et al., 2018; Lazarević et al. .2019).

While further investigation is desired to clarify the exact mechanism of atherosclerosis in CTX, accurate CVD risk assessment should be implemented to conduct preventive measures for risk reduction of atherosclerotic cardiovascular disease in these patients, as an opportunity to reduce the burden of CVD. Performing noninvasive evaluation of preclinical atherosclerosis may serve as an additional tool of risk stratification in CTX and, may assist in detecting the patients that will benefit from intensive lifestyle changes and appropriate pharmacotherapy.

## Data Availability

The raw data supporting the conclusion of this article will be made available by the authors, without undue reservation.

## Abbreviations

CDCA, chenodeoxycholic acid; CHD, coronary artery disease; cIMT, carotid intima-media thickness; CTX, cerebrotendinous xanthomatosis; CVD, cardiovascular disease; HDL, high-density lipoprotein; LDL, low-density lipoprotein; TC, total cholesterol; TG, triglycerides; GGT, gamma-glutamyltransferase; ALT, alanine aminotransferase; AST, aspartate aminotransferase; ALP, alkaline phosphatase.
